# Supplementary material for: The Case for Interpretable Geometry: Statistical Shape Models vs. Curvature-Based Descriptors in Aortic Disease Classification
Source: medRxiv. 2026 Jul 31:2026.07.29.26359299. Preprint. [Version 1] doi: 10.64898/2026.07.29.26359299 (PMC13484365; doi:10.64898/2026.07.29.26359299)
Supplement: Supplementary file 1 [file NIHPP2026.07.29.26359299v1-supplement-1.pdf]

medRxiv preprint doi: https://doi.org/10.64898/2026.07.29.26359299; this version posted July 31, 2026. The copyright holder has placed this preprint (which was not certified by peer review) in the Public Domain. It is no longer restricted by copyright. Anyone can legally share, reuse, remix, or adapt this material for any purpose without crediting the original authors.

Appendix A Supplementary Information

A.1 Landmarks sampling pipeline

The SSA pipeline described in Section 2.3 relies on a consistent parameterization of each aortic surface. Algorithm 1 details the procedure used to extract $N_s \times N_\theta$ landmark points per scan. At each centerline sample point, a cutting plane is defined by the local tangent vector, and the intersection curve with the aortic surface is resampled at equally spaced polar angles. The starting direction vector is propagated from one cross-section to the next to maintain angular consistency along the vessel.

medRxiv preprint doi: https://doi.org/10.64898/2026.07.29.26359299; this version posted July 31, 2026. The copyright holder has placed this preprint (which was not certified by peer review) in the Public Domain. It is no longer restricted by copyright. Anyone can legally share, reuse, remix, or adapt this material for any purpose without crediting the original authors.

Algorithm 1 Aorta's landmarks sampling

Choose a starting vector $\mathbf{v}_0 = [x, y, z]$
for each point P_i sampled from the centerline curve, $i = 1, \ldots, N_s$ **do**
    Find tangent $\mathbf{t}_i$ at P_i
    Set $\mathbf{n}_i \leftarrow \mathbf{t}_i$ and $O_i \leftarrow P_i$.
    Project $\mathbf{v}_{i-1}$ onto the plane orthogonal to $\mathbf{n}_i$ to obtain $\mathbf{v}_i = [x', y', z']$
    Set $r \leftarrow r_{\min}$. Generate a disk with normal $\mathbf{n}_i$, origin O_i and radius r
    while disk does not intersect the aorta **do**
        Increase r
    end while
    if disk intersects the aorta **then**
        Collect all intersection points $\{Q_j\}$
        if (gap between consecutive $Q_j >$ tolerance) **or** (intersection points do not form a closed loop) **then**
            Increase r
        else
            Resample N_θ points starting from the direction defined by $\mathbf{v}_i$ from the closed loop defined by $\{Q_j\}$
        end if
    end if
end for

A small number of cross-sections near the aortic arch produced incomplete intersection loops due to high local centerline curvature, which can cause the tangent plane to intersect branch vessels or miss the wall entirely. While noise doesn't contribute to the main principal components, such as PC_1 or PC_2, these misplaced landmark points might mix with subsequent principal components and affect classification results. For the results reported in this paper, affected landmarks are retained without manual correction; because PCA concentrates most variance in the leading components, isolated noisy landmarks have minimal impact on PC_1 and PC_2 but may contribute to the noise-dominated higher-order components discussed in Section 3.2.

A.2 Supplementary Classification Results

Figure A1 shows the distribution of the weighted F_1 score difference between SSA and geometric approach when applying to the same bootstrap samples drawn from the 290-scans cohort. Two GPCs are trained on either the first two principal components' score (SSA approach) or (R, $\widetilde{\delta K}$) and are evaluated on out-of-bag scans. Bootstrapping allows us to compare the two approaches under repeated resampling of the cohort, thereby quantifying the variability and uncertainty in their relative performance. On average, the geometric approach performs better than SSA approach, but this difference does not reach statistical significance, as the 95% confidence interval of the performance difference includes zero.

medRxiv preprint doi: https://doi.org/10.64898/2026.07.29.26359299; this version posted July 31, 2026. The copyright holder has placed this preprint (which was not certified by peer review) in the Public Domain. It is no longer restricted by copyright. Anyone can legally share, reuse, remix, or adapt this material for any purpose without crediting the original authors.

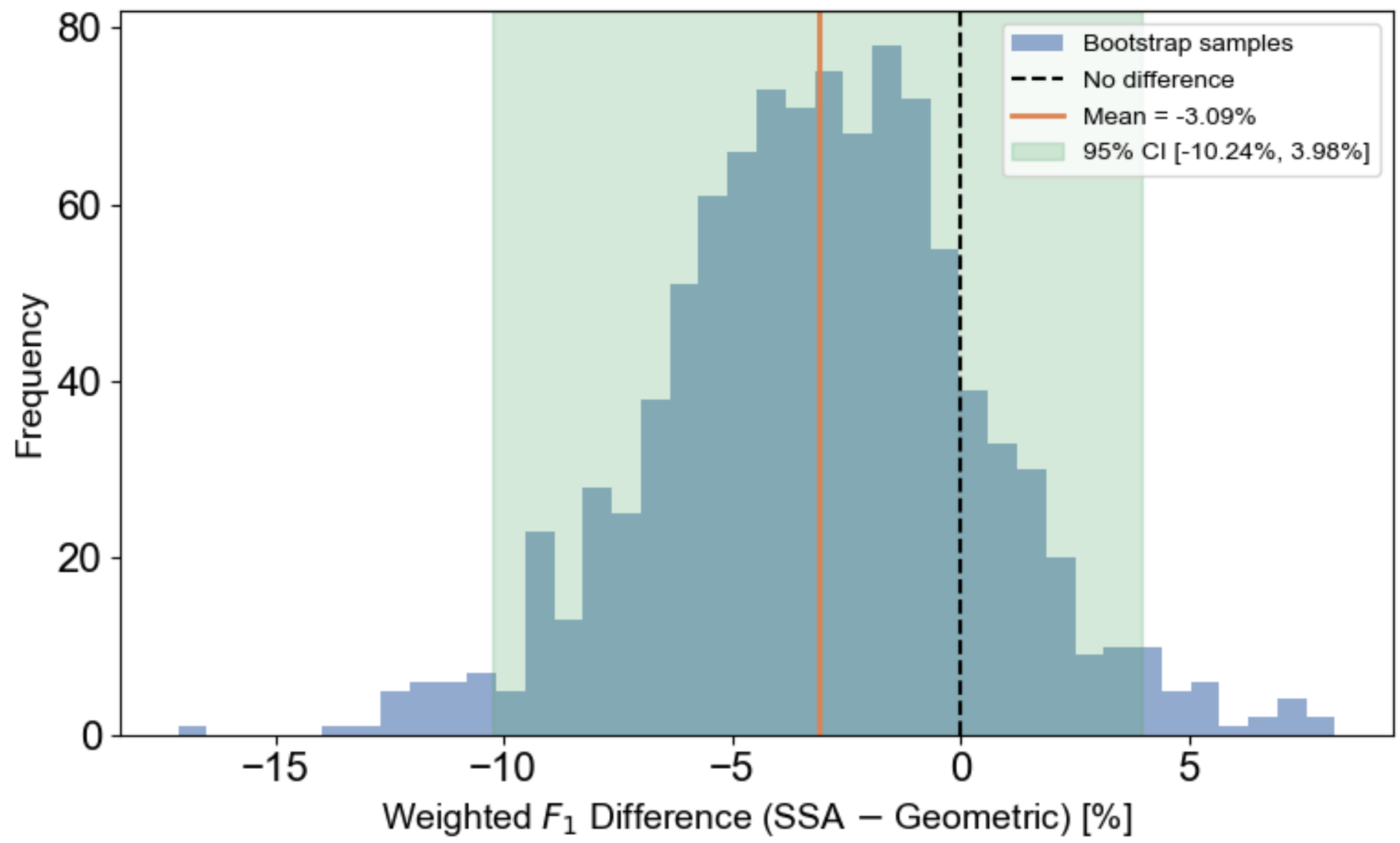

Fig. A1 Bootstrap distribution (1000 samples) of the GPC-predicted weighted F_1 score difference between SSA (2 features) and geometric approach, resampled from the 290-scan cohort. Positive values mean that SSA approach achieves a higher F_1 score. The dashed black line indicates the point where both approaches achieve equal performance, the orange vertical line indicates the mean difference, and the green shaded region shows 95% confidence interval. On average, the geometric approach achieves better F_1 score than SSA. However, this gain in performance by the geometric approach does not reach $p < 0.05$ significance level, as the 95% confidence interval passed the dashed black line.

Figure A2 presents the same head-to-head comparison shown in Figure 4, but using multinomial logistic regression in place of Gaussian process classification. With a linear decision boundary, the two approaches yield similar weighted F_1 scores (85.3% for SSA vs. 86.5% for the geometric descriptors), confirming that the performance gap between the methods is modest when classifier flexibility is limited. The advantage of the geometric feature space becomes more pronounced under the nonlinear GPC model (Figure 4), where the physically grounded axes allow the classifier to carve out distinct outcome regions that align with anatomical intuition.

medRxiv preprint doi: https://doi.org/10.64898/2026.07.29.26359299; this version posted July 31, 2026. The copyright holder has placed this preprint (which was not certified by peer review) in the Public Domain. It is no longer restricted by copyright. Anyone can legally share, reuse, remix, or adapt this material for any purpose without crediting the original authors.

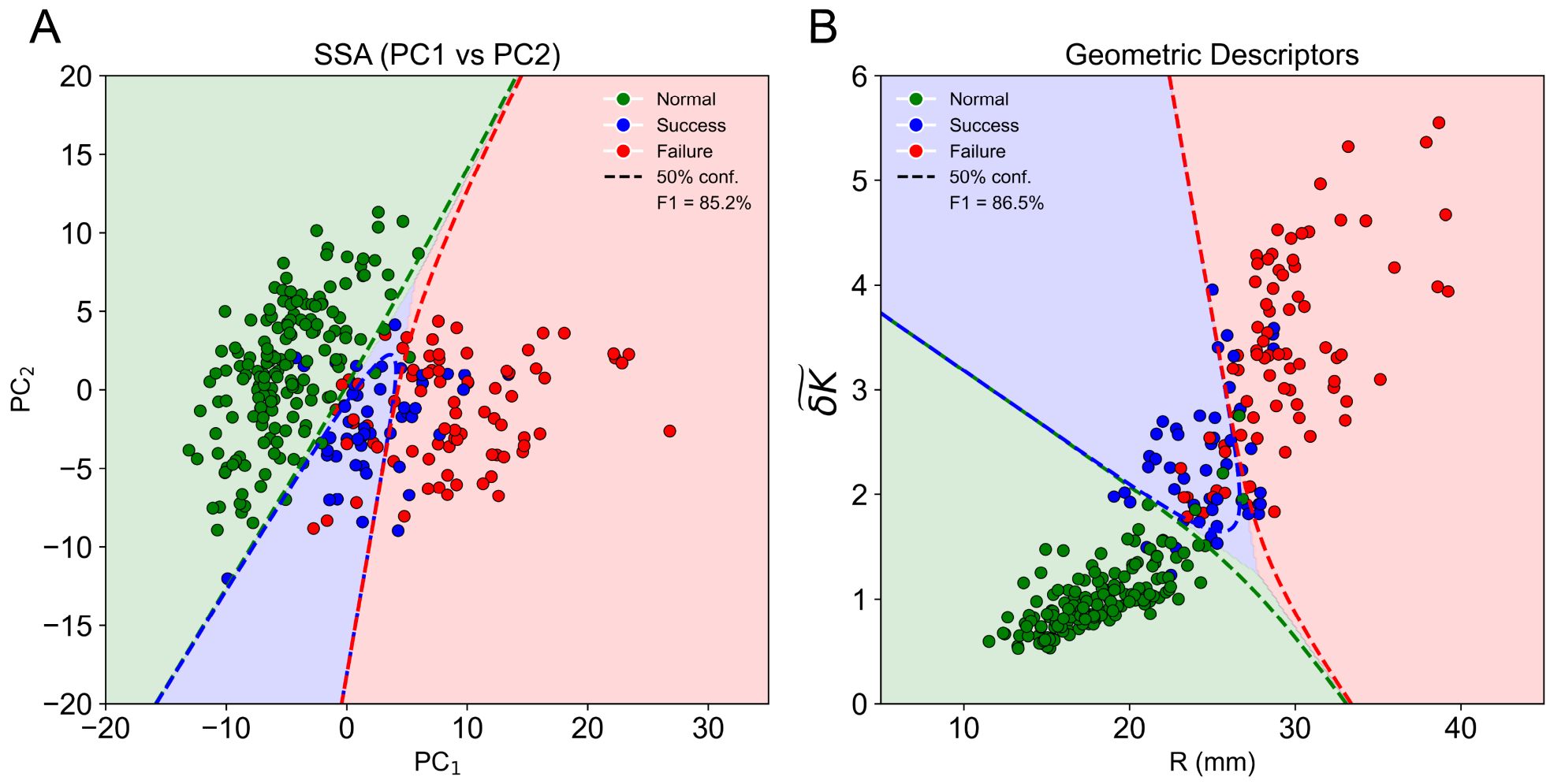

Fig. A2 Multinomial logistic regression decision boundaries. **(A)** SSA feature space (PC_1, PC_2) with linear decision boundaries from multinomial LR (weighted $F_1 = 85.2\%$). Shaded regions indicate predicted class territories; dashed lines denote the 50% probability contour for each class. **(B)** Geometric descriptor feature space (R vs. $\widetilde{\delta K}$) with multinomial LR boundaries (weighted $F_1 = 86.5\%$). The linear model in the geometric space yields comparable discrimination to SSA, with boundaries that are directly interpretable in terms of aortic size and surface irregularity. Green: Normal; blue: TEVAR Success; red: TEVAR Failure.

A.3 Mesh Alignment Quality

Reliable SSA requires that inter-subject differences in the landmark representation reflect genuine anatomical variation rather than residual misalignment. Figure A3 provides a more detailed view of the registration quality summarized in Figure 2**(A)**. At the tightest tolerance ($\tau = 0.01$), off-diagonal Surface DICE scores are uniformly low, as expected given true anatomical diversity across subjects. As the tolerance increases to $\tau = 0.1$, nearly all pairwise scores exceed 0.9, confirming that the Procrustes-based pipeline brings meshes into close spatial agreement at the scale relevant to our landmark sampling (cross-sectional spacing on the order of centimeters).

medRxiv preprint doi: https://doi.org/10.64898/2026.07.29.26359299; this version posted July 31, 2026. The copyright holder has placed this preprint (which was not certified by peer review) in the Public Domain. It is no longer restricted by copyright. Anyone can legally share, reuse, remix, or adapt this material for any purpose without crediting the original authors.

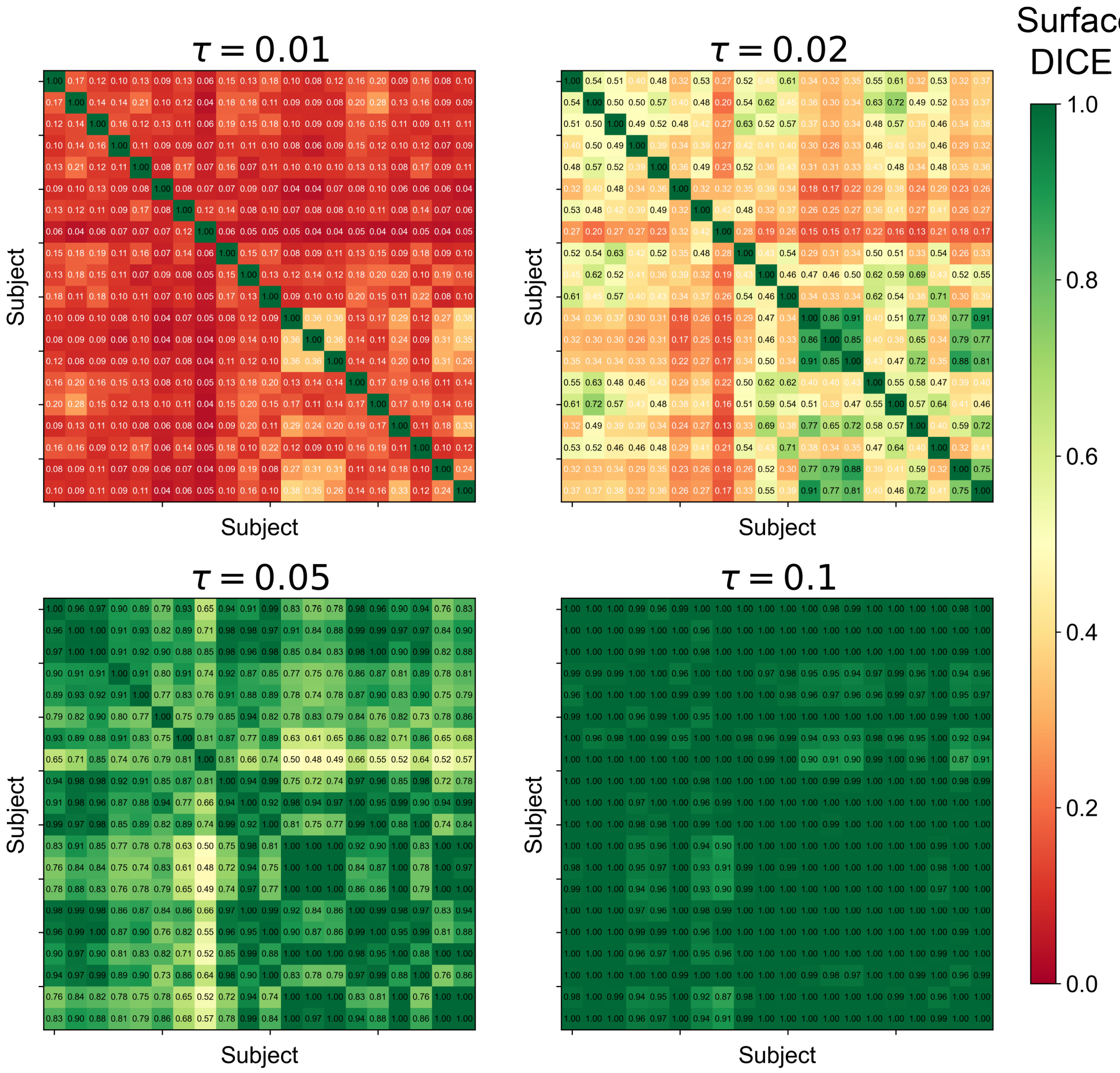

Fig. A3 Pairwise Surface DICE heatmaps across tolerance thresholds. Each panel shows the pairwise Surface DICE coefficient for a random subset of 20 aligned subjects at tolerance $\tau \in \{0.01, 0.02, 0.05, 0.1\}$. Meshes are subsampled to 1 000 points each. At tight tolerances ($\tau = 0.01$), off-diagonal scores are uniformly low, reflecting genuine inter-subject anatomical differences. As τ increases, the heatmap transitions toward uniformly high agreement ($\tau = 0.1$, nearly all entries ≥ 0.9), indicating that the Procrustes alignment successfully registers aortic surfaces to within this spatial tolerance. The diagonal is identically 1.0 by definition.

A.4 Principal Component Space Visualization

Figure A4 displays all six pairwise projections of the first four principal components extracted by the SSA pipeline. Consistent with the classification results in Section 3.1, the PC_1–PC_2 plane provides the clearest separation between normal and pathological groups, while higher-order components show substantial class overlap. This visualization motivates the focus on two-dimensional feature spaces throughout the main

medRxiv preprint doi: https://doi.org/10.64898/2026.07.29.26359299; this version posted July 31, 2026. The copyright holder has placed this preprint (which was not certified by peer review) in the Public Domain. It is no longer restricted by copyright. Anyone can legally share, reuse, remix, or adapt this material for any purpose without crediting the original authors.

text and supports the observation in Section 3.2 that retaining additional principal components beyond $N_p \approx 3$ contributes little generalizable discriminative information.

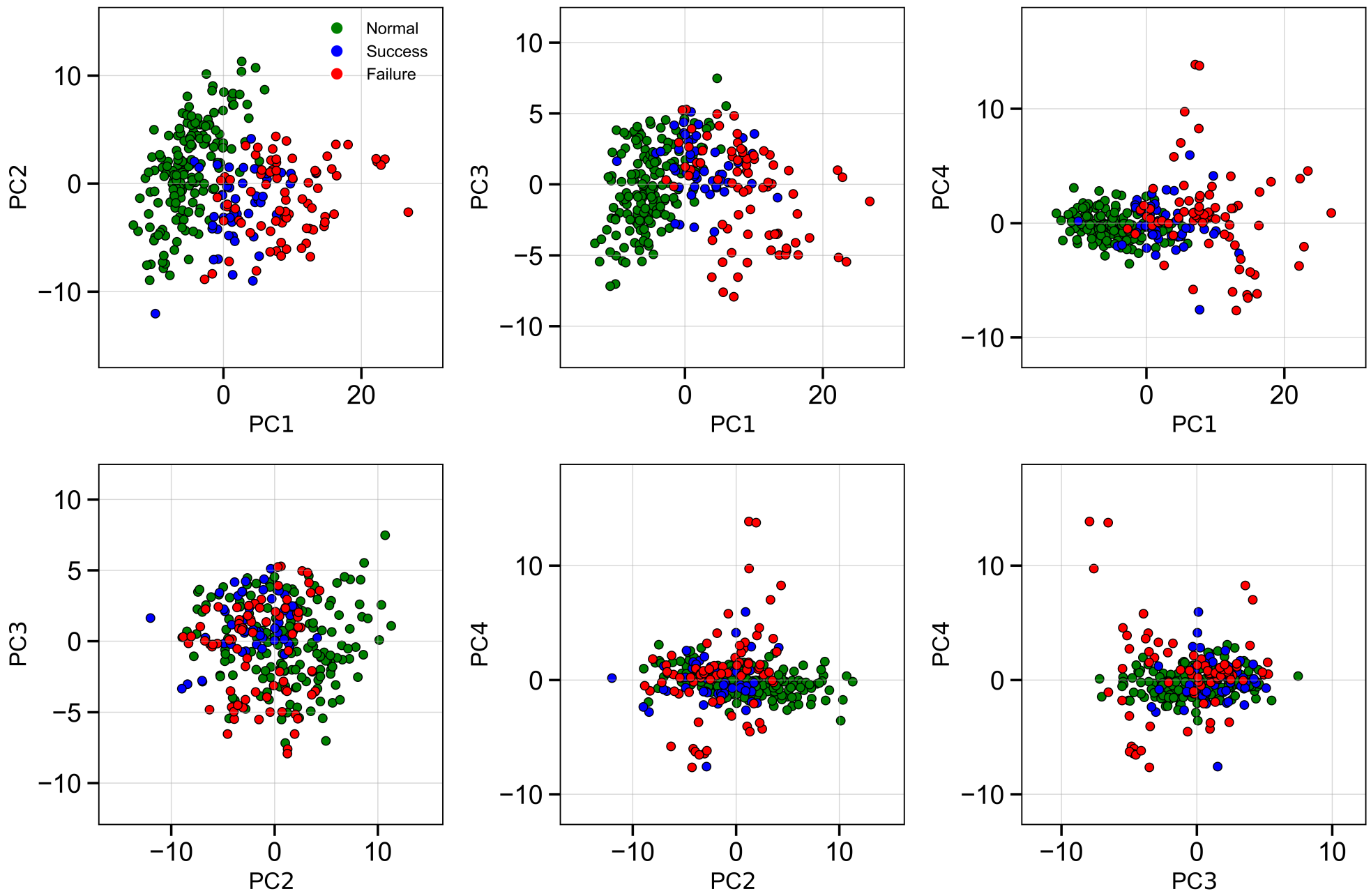

Fig. A4 Pairwise scatter plots of the first four principal components. All six pairwise projections of PC_1 through PC_4 from the SSA pipeline, colored by outcome class (green: Normal; blue: TEVAR Success; red: TEVAR Failure). The PC_1–PC_2 plane provides the strongest visual separation between normal and pathological groups, consistent with the dominance of these components in explaining overall data variance. Higher-order components (PC_3, PC_4) show substantially more class overlap, indicating that additional PCs contribute limited discriminative information.

A.5 Correlation Between Principal Components and Geometric Descriptors

A central question raised in Section 4.1 is whether the SSA feature space encodes the same anatomical information captured by the geometric descriptors. Figures A5 and A6 address this by plotting each of the first four principal components against mean aortic radius and $\widetilde{\delta K}$, respectively.

PC_1 is strongly correlated with both R ($r = 0.80$) and $\widetilde{\delta K}$ ($r = 0.83$), confirming that the dominant mode of shape variation captured by PCA is largely co-linear with the size–shape axis that defines the geometric feature space. PC_2 shows moderate inverse correlations with both descriptors ($r = -0.44$ for R, $r = -0.36$ for $\widetilde{\delta K}$), suggesting that it captures a mixture of size and shape variation that cannot be cleanly attributed to either quantity alone. PC_3 and PC_4 are effectively uncorrelated with both geometric descriptors, indicating that higher-order shape modes encode

medRxiv preprint doi: https://doi.org/10.64898/2026.07.29.26359299; this version posted July 31, 2026. The copyright holder has placed this preprint (which was not certified by peer review) in the Public Domain. It is no longer restricted by copyright. Anyone can legally share, reuse, remix, or adapt this material for any purpose without crediting the original authors.

anatomical variation—or noise—that is orthogonal to the clinically relevant size and curvature heterogeneity captured by R and $\widetilde{\delta K}$.

Taken together, these results demonstrate that the discriminative information in the SSA manifold is largely redundant with the two geometric descriptors, which carry the additional advantage of direct physical interpretability.

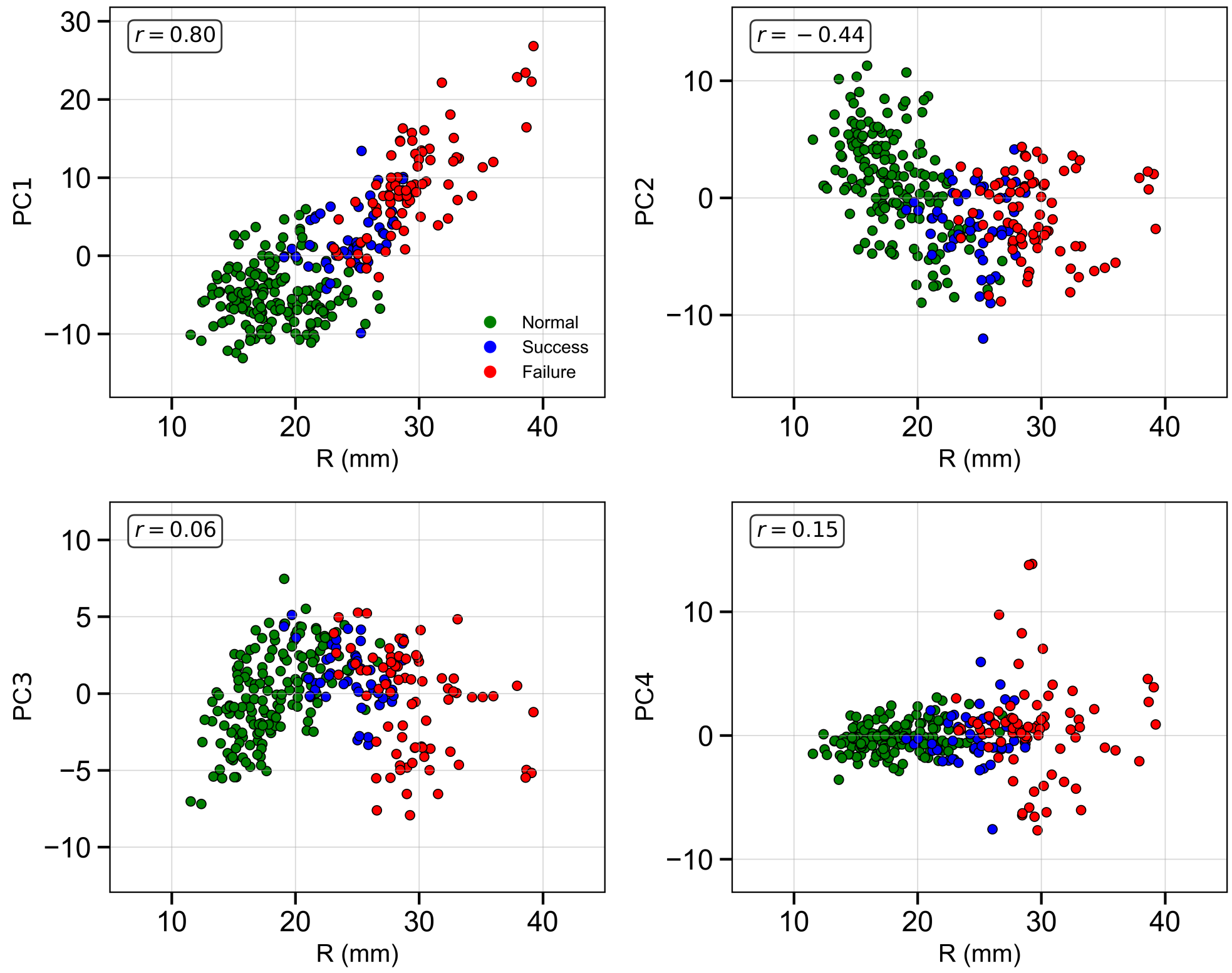

Fig. A5 Correlation between SSA principal components and mean aortic radius. Scatter plots of mean aortic radius (mm) versus each of the first four principal components, colored by outcome class. Pearson correlation coefficients are shown inset. PC_1 is strongly correlated with mean radius ($r = 0.80$), confirming that the leading mode of shape variation captured by SSA predominantly reflects overall aortic size. PC_2 shows a moderate inverse correlation ($r = -0.44$), while PC_3 and PC_4 are effectively uncorrelated ($r = 0.06$ and $r = 0.15$, respectively).

medRxiv preprint doi: https://doi.org/10.64898/2026.07.29.26359299; this version posted July 31, 2026. The copyright holder has placed this preprint (which was not certified by peer review) in the Public Domain. It is no longer restricted by copyright. Anyone can legally share, reuse, remix, or adapt this material for any purpose without crediting the original authors.

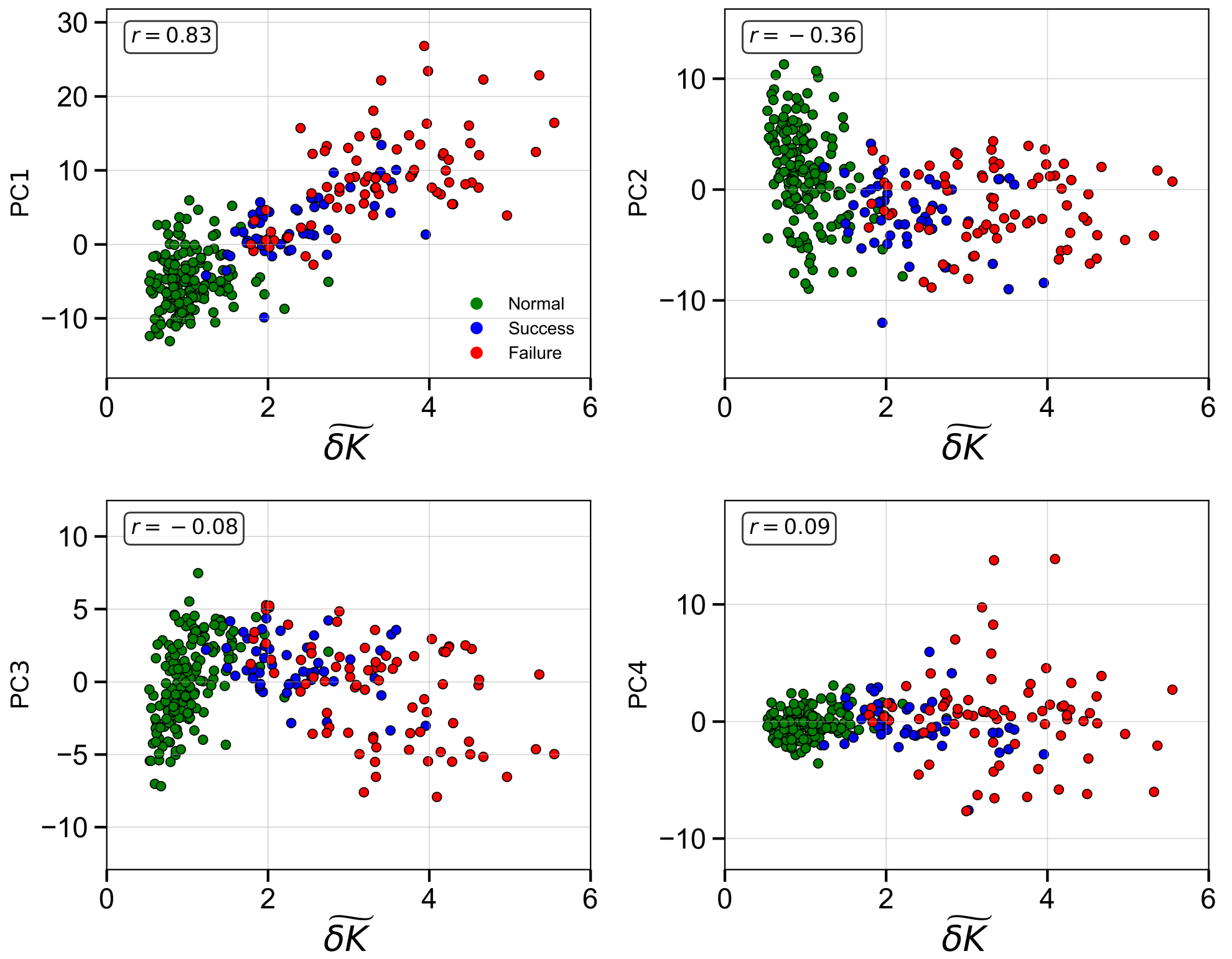

Fig. A6 Correlation between SSA principal components and Gaussian curvature fluctuation. Scatter plots of normalized Gaussian curvature fluctuation ($\widetilde{\delta K}$) versus each of the first four principal components, colored by outcome class. PC_1 exhibits a strong positive correlation with $\widetilde{\delta K}$ ($r = 0.83$), indicating that the dominant mode of variation tracked by SSA is roughly co-linear with the curvature-based geometric descriptor. PC_2 shows a moderate negative correlation ($r = -0.36$), while PC_3 and PC_4 are essentially uncorrelated ($r = -0.08$ and $r = 0.09$). Together with Figure A5, these results demonstrate that much of the discriminative information in the SSA manifold is redundant with the two geometric descriptors (R and $\widetilde{\delta K}$), which carry the additional advantage of direct clinical interpretability.
